# Randomized double-blind clinical trial comparing two anesthetic techniques for ultrasound-guided transvaginal follicular puncture

**DOI:** 10.1590/S1679-45082016AO3714

**Published:** 2016

**Authors:** Gilvandro Lins de Oliveira, Fernando Cesar Serralheiro, Fernando Luiz Affonso Fonseca, Onésimo Duarte Ribeiro, Fernando Adami, Denise Maria Christofolini, Bianca Bianco, Caio Parente Barbosa

**Affiliations:** 1 Faculdade de Medicina do ABC, Santo André, SP, Brazil

**Keywords:** Anesthesia, Reproductive techniques, Fentanyl, Propofol, Midazolam

## Abstract

**Objective:**

To compare the anesthetic techniques using propofol and fentanyl *versus* midazolam and remifentanil associated with a paracervical block with lidocaine in performing ultrasound-guided transvaginal oocyte aspiration.

**Methods:**

A randomized double-blind clinical trial (#RBR-8kqqxh) performed in 61 women submitted to assisted reproductive treatment. The patients were divided into two groups: anesthetic induction with 1mcg/kg of fentanyl associated with 1.5mg/kg of propofol (FP Group, n=32), in comparison with anesthetic induction using 0.075mg/kg of midazolam associated with 0.25mcg/kg/min of remifentanil, and paracervical block with 3mL of 2% lidocaine (MRPB Group, n=29). Main outcome measures: human reproduction outcomes, modified Aldrete-Kroulik index, hemodynamic parameters, and salivary cortisol.

**Results:**

The results revealed a higher number of embryos formed in the FP Group (p50=2 *versus* 1; p=0.025), gestation rate two times higher in the FP Group (44.4% *versus* 22.2%; p=0.127), less time to reach AK=10 in the MRPB Group (p50=10 *versus* 2; p<0.001), and lower mean of hemodynamic parameters in the MRPB Group (p<0.05).

**Conclusion:**

Anesthesia with fentanyl and propofol as well as with midazolam, remifentanil, and paracervical block offered satisfactory anesthetic conditions when performing assisted reproduction procedures, providing comfort for the patient and physician.

## INTRODUCTION

Follicular aspiration is one of the steps of assisted reproductive treatment, and is considered the most painful procedure for patients due to puncture of the ovarian capsule and manipulation of the needle in the pelvis.^(1)^ Analgesia should be offered for the procedure for less discomfort and lower risk of complications. The choice of anesthetics should respect the potential risks of these drugs for the reproductive results.^(2)^

Anesthetics show systemic activity, reach the ovaries, and can cause harmful effects, possibly by the accumulation of these substances in the follicular fluid. The use of propofol in assisted reproduction procedures has already been established, and the concentration of this medication in follicular fluid is proportional to the total quantity infused, since high doses are needed to reach effects that harm oocyte quality. Thus, prolonged procedures with greater quantities of anesthetics could affect quality of oocytes.^(3,4)^ The combined use of fentanyl and propofol enables decreasing the maintenance dose of the second drug, with an adequate anesthesia plan and shorter time for recovery of consciousness.^(5)^ Remifentanil has properties appropriate for anesthesia at outpatient´s setting, such as rapid onset, short half-life even with continuous infusion, and potent analgesia, and can be used alone or as an adjuvant drug.^(6,7)^ Remifentanil associated with paracervical block allows satisfactory analgesia and sedation for follicular puncture.^(8)^ Various techniques have been described in literature for promoting a safer and more comfortable ovarian puncture, from local block to general anesthesia. Nevertheless, there is no literature confirming the superiority of one technique over the others.

## OBJECTIVE

To compare techniques of anesthesia with propofol and fentanyl *versus* midazolam and remifentanil, associated with paracervical block with lidocaine, in ultrasound-guided aspiration of oocytes.

## METHODS

### Patients

This is a randomized double-blind clinical trial clinical trial number RBR-8kqqxh, carried out in 61 women, classified according to the scale of the American Society of Anesthesiologists (ASA), as ASA I and II, aged under 42 years, body mass index (BMI) under 30, with a diagnosis of infertility according to the work-up for infertile couples seen at the Center for Human Reproduction and Genetics of the *Faculdade de Medicina do ABC.* They were submitted to highly complex assisted reproductive treatment during the period March 2012 to June 2013. The patients were randomly allocated to two groups by means of a simple drawing, and the allocator and patients were blinded. Randomization was performed by an investigator responsible for randomly selecting the patient group in which the individual would be placed: fentanyl, propofol (FP Group), and midazolam, remifentanil and paracervical block (MRPB Group). This was carried out with a draw to define the group selected. Neither the allocator nor the patients knew the correlation. All patients presented with at least one ovarian follicle larger than 17mm to be retrieved. The first group of 32 patients was submitted to anesthetic induction with 1mcg/kg of fentanyl associated with 1.5mg/kg of propofol (FP Group); for maintenance, fractioned bolus of 20mg of propofol was used as required for each patient, taking into consideration the experience of the anesthesiologist. In the second group, 29 patients were submitted to anesthetic induction with 0.075mg/kg of midazolam associated with 0.25mcg/kg/minute of remifentanil (MRPB Group), with addition of 0.05mcg/kg/minute of remifentanil in cases of complaints of pain or patient movement, and decreased by 0.05mcg/kg/minute in cases of respiratory rate under 8 breaths per minute (bpm), heart rate under 45 beats per minute (bpm), and peripheral oxygen saturation (SpO_2_) under 92%. In this same group, a paracervical block was also done with 3mL of 2% lidocaine, and the blocking points were 3 and 9 o'clock.

Clinical data and saliva samples were only collected after explanation of the objective of the study and signing of the Informed Consent Form, which was approved by the local Research Ethics Committee, CAAE: 10163812.5.0000.0082, protocol number 164.518.

### Anesthesia

All patients were submitted to a pre-anesthetic visit and monitoring with a cardioscope, non-invasive arterial pressure monitoring, and pulse oximetry. Parameters were measured and recorded at the onset of anesthesia and every 5 minutes. Baseline data were documented upon patient's admission. Oxygen was administered with a nasal catheter, and in the case of SpO_2_ equal to or less than 92%, the oxygen facial mask was used with the purpose of assisted breathing, if needed.

### Salivary cortisol

**Figure 1:**
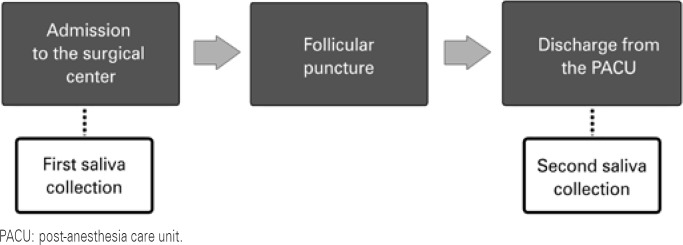
Flowchart of cortisol collection times

Collection of saliva was performed at two different times: first, before the procedure, upon admission of patient to the operating room, and the second, at the time of discharge from the post-anesthesia care unit, using Salivette^®^ tubes (Sarstedt AG & Co, Nümbrecht, Germany) (Figure 1). Next, the samples were centrifuged at 3,000 revolutions per minute (rpm) for 15 minutes and stored at -20°C. Dosing was performed using a commercial kit (Cortisol, DiaMed^®^, FRA) that uses the colorimetric competitive immune enzyme assay method, with Labortech equipment. Analyses were carried out following good clinical analysis practices.

### Statistical analysis

To calculate the sample size, the standard deviation value of 5.4 punctured follicles was defined; the difference to be detected was 4.3 punctured follicles; the significance level was 5%, and a power of 80%, with a loss of approximately 10%, according to Hammadeh et al.^(9)^ Based on these parameters, we ended up with 30 women per group.

Bearing in mind the non-normality of the variable quantitative data, it was decided to describe them based on values of medians, 25^th^, and 75^th^ percentiles, with Mann-Whitney's hypothesis test. Qualitative variables were described by means of absolute and relative frequencies, using the χ^2^ test to analyze association. Statistical software used was Stata^®^ 11.0 (Chicago, IL, USA). We considered as statistically significant a p value <0.05.

## RESULTS

A total of 62 women were screened for the study, but 1 was excluded for having been treated to donate the oocytes collected, and not for reproductive purposes. Both groups were harmonized, with no significant difference as to age of 35 (32.5 to 38) *versus* 36 (33 to 40) years (p=0.433); BMI of 22.7 (21.6 to 25.3) *versus* 23.4 (22.1 to 25) (p=0.778); male infertility factor 18 *versus* 14 (p=0.533); time of infertility 3 *versus* 3 years (p=0.710); ASA I/II classification (p=0.649) 20 *versus* 12 prior treatments (p=0.099); dose of 100 or 200IU of recombinant follicle stimulating hormone (rFSH) per day (p=0.986); number of follicles more than 14mm in diameter visualized by ultrasound-guided (USG) as 5 *versus* 4 (p=0.315); and fertilization technique performed by intracytoplasmic sperm injection (ICSI) *versus* in vitro fertilization/intracytoplasmic injection of sperm (IVF/ICSI) (p=0.527) (Table 1).

**Table 1:** Description of baseline variables of the women submitted to assisted reproduction technique, per type of anesthesia

Variables		Anesthesia technique		p value*
		FP Group (n=32)	MRPB Group (n=29)	
Median†				
Age, years		35 (32.5;38)	36 (33;40)	0.443
Time of infertility, years		3 (1.5;5)	3 (2;5)	0.710
BMI, kg/m^2^		22.7 (21.6;25.3)	23.4 (22.1;25)	0.778
Number of follicles by USG		5 (3;9)	4 (3;9)	0.315
ASA status, n (%)				
	I	25 (78.1)	24 (82.8)	0.649
	II	7 (21.9)	5 (17.2)	
Prior assisted reproductive treatments		20 (62.5)	12 (41.4)	0.099
Dose of rFSH/day, n (%)				
	100IU	10 (31.2)	9 (31.0)	0.986
	200IU	22 (68.7)	20 (69.0)	
Oocyte fertilization technique, n (%)				
	With no oocyte to be fertilized	3 (9.4)	4 (13.8)	0.527
	ICSI	26 (81.2)	20 (69.0)	
	IVF/ICSI	3 (9.4)	5 (17.2)	
Male factor, n (%)		18 (56.3)	14 (48.3)	0.533
Age >40 years		6 (18.7)	6 (20.7)	0.849
Endometriosis, n (%)		5 (15.6)	3 (10.3)	0.542
Tubal factor, n (%)		5 (15.6)	6 (20.7)	0.607
Infertility with no known cause, n (%)		6 (18.7)	3 (10.3)	0.355

* Mann-Whitney test for quantitative variables and χ^2^ test for qualitative variables;

† p25 and p75: 25^th^ and 75^th^ percentiles.

FP Group: fentanyl and propofol group; MRPB Group: midazolam, remifentanil and paracervical block group; BMI: body mass index; USG: ultrasound; ASA: American Society of Anesthesiologists; rFSH: recombinant follicle stimulating hormone; ICSI: intracytoplasmic injection; IVF: *in vitro* fertilization.

The primary endpoints were results for assisted reproduction. No statistical significance was found as to the number of follicles punctured: 5 (3;9) *versus* 3 (2;7) (p=0.212), number of oocytes in metaphase II (MII) 4.5 (2;7.5) *versus* 3 (2;6) (p=0.210), or rate of fertilized oocytes 66.8 (50;100) *versus* 50 (0;83.3) (p=0.061). For the number of embryos formed, there was a statistically significant difference, namely 2 (2;4) *versus* 1 (0;3) (p=0.025), and for the rate of gestation, it was noted that the rate of the FP Group was twice that of the MRPB Group, 44.4% *versus* 22.2% (p=0.127) (Table 2).

**Table 2:** Description of outcomes related to reproductive health of the women submitted to the assisted reproductive technique per type of anesthesia

Variables		Anesthesia technique		p value*
		FP Group (n=32)	MRPB Group (n=29)	
Median†				
	Number of follicles in USG	5 (3;9)	3 (2;7)	0.212
	Number of oocytes in MII	4.5 (2;7.5)	3 (2;6)	0.210
	Rate of fertilization	66.8 (50;100)	50 (0;83.3)	0.061
	Number of embryos	2 (2;4)	1 (0;3)	0.025
	Rate of pregnancy n (%)	44.4	22.2	0.127

* Mann-Whitney test for quantitative variables and χ^2^ test for qualitative variables;

† p25 and p75: 25^th^ and 75^th^ percentiles; sample size of the FP Group: 27; sample size of the MRPB Group: 18.

FP Group: fentanyl and propofol group; MRPB Group: midazolam, remifentanil and paracervical block group; USG: ultrasound.

There were 5 exclusions in the FP Group due to the following reasons: no embryo for transfer (n=1), no oocytes in MII (n=2), and frozen embryos (n=2), and 11 exclusions in the MRPB Group due to the following reasons: no embryo for transfer (n=7), no oocytes in MII (n=3), and vitrified oocytes (n=1).

As to the anesthesia data and secondary endpoints, statistical significance was found as to time to awaken after the end of the procedure, using as reference the modified Aldrete-Kroulik (AK) index (1995).^(10)^ The AK index was evaluated at 2 minutes 7 (7;8) *versus* 10 (9;10) and at 5 minutes 8 (8;9) *versus* 10 (10;10); in addition to the time to reach an AK index equal to 10 10 (8,5;10) *versus* 2 (1;4), all with p<0.001, with clinical importance for ambulatory anesthesia, respectively for FP Group and MRPB Group. The same was true for the average of mean arterial pressure levels in mmHg (76.5 *versus* 70; p=0.003), heart rate in bpm (79.3 *versus* 71; p=0.008) and SpO_2_ (98.5% *versus* 99%; p=0.004), but with no clinical significance.

Also used was the patient's evaluation of pain after awaking using a verbal number scale for pain between zero and 10, with results of 2 *versus* 0 (p<0.01), the degree of satisfaction as to the anesthetic procedure between zero and 10 with values of 10 (9;10) *versus* 10 (9;10) (p=0.956), and the presence of nausea/vomiting in the immediate postoperative period, with results of 2 (6.3%) *versus* 1 (3.5%) (p=0.613) (Table 3).

**Table 3:** Description of outcomes related to anesthesia of the women submitted to the assisted reproduction technique per type of anesthesia

Variables	Anesthesia technique		p value*
	FP Group (n=32)	MRPB Group (n=29)	
Median†			
AK score‡ at 2 minutes	7 (7;8)	10 (9;0)	<0.001
AK score‡ at 5 minutes	8 (8;9)	10 (10;0)	<0.001
Time to reach an AK score‡ equal to 10, minutes	10 (8.5;10)	2 (1;4)	<0.001
Mean heart rate	79.3 (71.9;85.8)	71 (67;78)	0.008
Mean blood pressure, mmHg	76.5 (72.4;81.6)	70 (65;76)	0.003
Mean peripheral oxygen saturation, %	98.4 (91.1;99)	99 (98.4;100)	0.004
Pain	2 (1.5;5)	0 (0;0)	<0.001
Patient satisfaction level	10 (9;10)	10 (9;10)	0.956
Difference in cortisol	-0.6 (-2.3;0.5)	0.7 (-0.6;1.8)	0.061
Nausea, n (%)	2 (6.3)	1 (3.5)	0.613

* Mann-Whitney test for quantitative variables and χ^2^ test for qualitative variables;

† p25 and p75: 25^th^ and 75^th^ percentiles;

‡ Aldrete-Kroulik Index.^(10)^

FP Group: fentanyl and propofol group; MRPB Group: midazolam, remifentanil and paracervical block group; AK: Aldrete-Kroulik.

Analysis of salivary cortisol concentration was performed based on the measurement of the difference between salivary cortisol 30 minutes after the end of the procedure and the baseline value, considering what was obtained before the surgical procedure. All collections were performed during the morning period, thus avoiding the effect of the circadian cycle. The difference between salivary cortisol dosing (30 minutes after awaking and immediately before the procedure) showed a tendency towards statistical significance (p=0.061), and was greater in the group with FP anesthesia.

## DISCUSSION

This prospective randomized study had the objective of comparing two anesthetic techniques: propofol and fentanyl with remifentanil, midazolam, and paracervical block in USG-guided transvaginal follicular puncture. The groups were paired as to demographic and clinical data. This study evaluated the reproductive results, including the number of punctured follicles, number of oocytes with maturation degree at MII, rate of fertilized oocytes, number of formed embryos, as well as the rate of gestation. The results suggest a greater tendency towards gestation in the group submitted to anesthesia with FP, with a rate two times higher relative to the group of anesthesia with MRPB; this tendency also occurred in the number of oocytes fertilized. The findings also suggested a greater number of embryos formed in the FP Group.

Bümen et al.^(11)^ compared two different anesthetic techniques in 70 patients submitted to USG-guided transvaginal follicular puncture, using general anesthesia with propofol associated with remifentanil, and maintenance conducted with propofol in bolus according to the patient's need, in comparison with the group submitted to paracervical block with 2% prilocaine associated with intramuscular meperidine. The study demonstrated that in the general anesthesia group, there was a higher rate of pregnancy (56.3% *versus* 44.7%; p=0.47) and a greater number of embryos transferred (2.7 *versus* 2.4; p=0.045), with data that is similar to that found in the present study.^(11)^

The present study was also aimed to compare the intraoperative anesthetic quality between the groups, assessing mean heart rate, mean blood pressure, and mean SpO_2_ in which all these variables showed lower values in the MRPB Group. Taking into consideration that the procedures of assisted reproduction require outpatient-type anesthesia in which the patient will be discharged from the hospital on the same day, we evaluated the time to awake the patients in both groups, analyzing the following criteria: AK score value at 2 and at 5 minutes, and the time in minutes necessary to reach an AK 10 score. Higher scores were found with a smaller time interval in the MRPB Group, suggesting that anesthesia allows earlier awakening of the patients in this group. Its use is appealing for short-duration procedures, since it allows earlier hospital discharge of the patients and better surgical flow.

The intensity of pain was measured when patients were awake, and the verbal numeric scale was used, ranging from zero to 10, in which zero is no pain and 10 is the greatest pain ever felt. The numbers were smaller in the MRPB Group, likely suggesting more adequate pain control, which might be attributed to the paracervical block.

More recently, Coskun et al.^(6)^ assessed anesthetic techniques in 69 women with continuous infusion of propofol associated with remifentanil, and continuous infusion divided into three groups according to the controlled target infusion of remifentanil of 1.5, 2.0, and 2.5ng/mL.^(6)^ The techniques provided conditions adequate for the procedure in all groups.

The present article evaluated the variation of salivary cortisol concentration, for being a good marker of response to stress.^(12)^ The concentration was dosed before the procedure (baseline concentration) and 30 minutes after the procedure ended, with the purpose of evaluating the influence of cortisol (a hormone present in the metabolic response to the aggressor agent) on the reproductive outcome. Salivary cortisol was chosen since it has been shown to be a precise indicator of total plasma cortisol and free plasma cortisol, besides being more easily collected without aggressive procedures.^(13)^ All samples were collected in the morning, to exclude the effect of the circadian cycle, and it was noted that the variation in the FP Group was -0.6ng/mL *versus* 0.7ng/mL in the MRPB Group. Based on these findings, in the first group, the second dosing was lower than the first, whereas the opposite occurred in the second group. The group with the lowest response to stress and variation of salivary cortisol seems to have had a better reproductive outcome, that is, a higher pregnancy rate.

An et al.^(14)^ conducted a study in 264 patients submitted to the first assisted reproductive procedure in order to evaluate the response of the sympathetic nervous system with reproductive outcome, assessed by dosing cortisol and noradrenaline serum levels in plasma and follicular fluid during oocyte retrieval. It was demonstrated that the levels of cortisol and noradrenaline serum levels were low in the group of pregnant women, with statistical significance (p<0.001).^(14)^ This finding shows an influence of the acute response to stress as a harmful factor for the reproductive outcome, corroborating the findings of the present study.

Azemati et al.^(15)^ measured hormone and metabolic changes in one hundred women submitted to gynecological laparoscopy. The study showed that in the propofol and remifentanil group, cortisol showed a significant decrease one hour after the beginning of surgery.^(15)^ A similar finding was identified in the present study, *i.e*., likely the propofol decreases the metabolic response to trauma and is beneficial to the reproductive outcomes.

## CONCLUSION

In synthesis, anesthesia with fentanyl and propofol, as well as with midazolam, remifentanil and a paracervical block offer satisfactory anesthetic conditions for carrying out ultrasound-guided transvaginal follicular puncture, providing comfort to the patient and the physician. The interference of the anesthetic choice in the reproductive outcome still requires further studies with larger population groups, but this study showed that the use of propofol with fentanyl has a tendency to improve reproductive outcome with improvement in the number of embryos, as well as a higher fertilization rate, probably by decreasing the elevation of stress hormones during the procedure.
